# “In front of patients, I will always be a pupil.” Dr. Xiaoqian Zhang: the founder of the modern Chinese gastroenterology

**DOI:** 10.1007/s13238-018-0561-4

**Published:** 2018-07-18

**Authors:** Lu Wang, Xudong Liu, Wenli Duan, Shu-yang Zhang

**Affiliations:** Peking Union Medical College Hospital, Chinese Academy of Medical Sciences and Peking Union Medical College, Beijing, 100730 China

Dr. Xiaoqian Zhang (张孝骞, Hsiao-Chien Chang) was an outstanding clinician, therapist and medical educator, a member of the Academic Divisions of the Chinese Academy of Sciences, he is considered to be the founder of the modern Chinese gastroenterology (PUMCH, 1988). As a doctor, he made a systemic and deep research on human blood volume, gastric secretion function, peptic ulcer disease, gastric ulcer and gastric cancer, celiac tuberculosis, amoebic dysentery, ulcerative colitis and so on. He diagnosed and treated multiple intractable diseases. As an educator, he emphasized the training of clinical basic skills, urged students to grasp the utilization of science in study and work. He trained a large number of talents for Chinese medicine.

**Figure 1:**
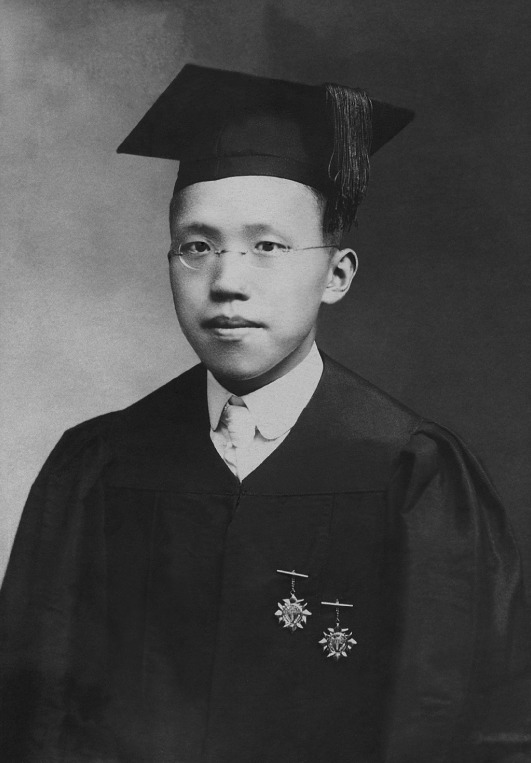
**Dr. Xiaoqian Zhang (Dec. 28th, 1897–Aug. 8th, 1987)**

Dr. Zhang was born on December 28, 1897, in a family of teachers, Changsha, Hunan Province. In 1914, he chose to enter Hsiang-Ya Medical College studying medicine, for he realized that “poverty” which leaned to industrial salvation was not the only contradiction at that time, the destructiveness of “disease” was as deep as it was (Zhang, 1983). Under the influence of William Osler’s story, the father of Modern Medicine, Dr. Zhang had been indulging in the extensive and profound internal medicine research field for a few decades and had struggled for a lifetime. In 1921, he graduated from Hsiang-Ya Medical College and received his M.D. from the Connecticut government (Fig. 1). After that, he became a resident doctor in Hsiang-Ya Medical College (PUMCH, 1988).

In 1923, he was offered a chance to further study at Peking Union Medical College Hospital (PUMCH). During the residency period in PUMCH, because of his excellent performance, he got an opportunity to study in the Johns Hopkins Hospital for one year in 1927. Under Prof. Harrop’s supervision, he immersed in the study of human blood volume. In 1928, he published two research articles in the *Journal of Clinical Investigation* on progress in blood volume, which was highly appraised by peer scientists and had been widely referenced in professional literature throughout the world (Chang and Harrop, 1928; Chang et al., 1928). Though being invited to stay in the United States, he was concerned more about his impoverished motherland. After returning to China, he hosted a series of research work. In 1931, he published another research article entitled *the blood volume in hyperthyroidism* on *Journal of Clinical Investigation*, which was also received extensive attention from peer scientists worldwide (Chang, 1931). In the 1930s, he created the first gastroenterology group in PUMCH and published several research articles on gastric secretion function (Chang, 1933). Since then, Dr. Zhang developed into a well-known clinician gradually.

After the “September 18th Incident”, the Japanese aggressors pressed in step by step. Dr. Zhang, who had been working in PUMCH for 13 years at that time, decided not to work under the control of the invaders. In 1937, he accepted the invitation from his alma mater and moved back to Hsiang-Ya Medical College to continue his study. Unfortunately, as the Japanese aggressors advanced southward, Hsiang-Ya could no longer survive in Changsha. Teachers and students left school one after another, and the college was deep in crisis. At the most dangerous moment, Dr. Zhang took over the role of dean and decided to move the whole college westward to Guiyang. They overcame various difficulties, taking 40 tons of teaching equipment and books with them. Students, faculty staff and their families, approximately 300 people, finally reached Guiyang in one week. In the winter of 1944, the Japanese invaded Guiyang and the college was in panic again. At that time, Dr. Zhang once again led the teachers and students to set foot on a long journey to Chongqing and continued their classes until the end of the War (Rong and Liu, 1987). With perseverance and courage, he avoided the Hsiang-Ya from being destroyed by war and reserved an important source for the Chinese medical science, which was a vivid interpretation of his patriotic feelings that “spring of life, even if mixed with blood and tears, should flow in one’s own country” (PUMCH and Xiangya Hospital of Central South University, 2008).

**Figure 2:**
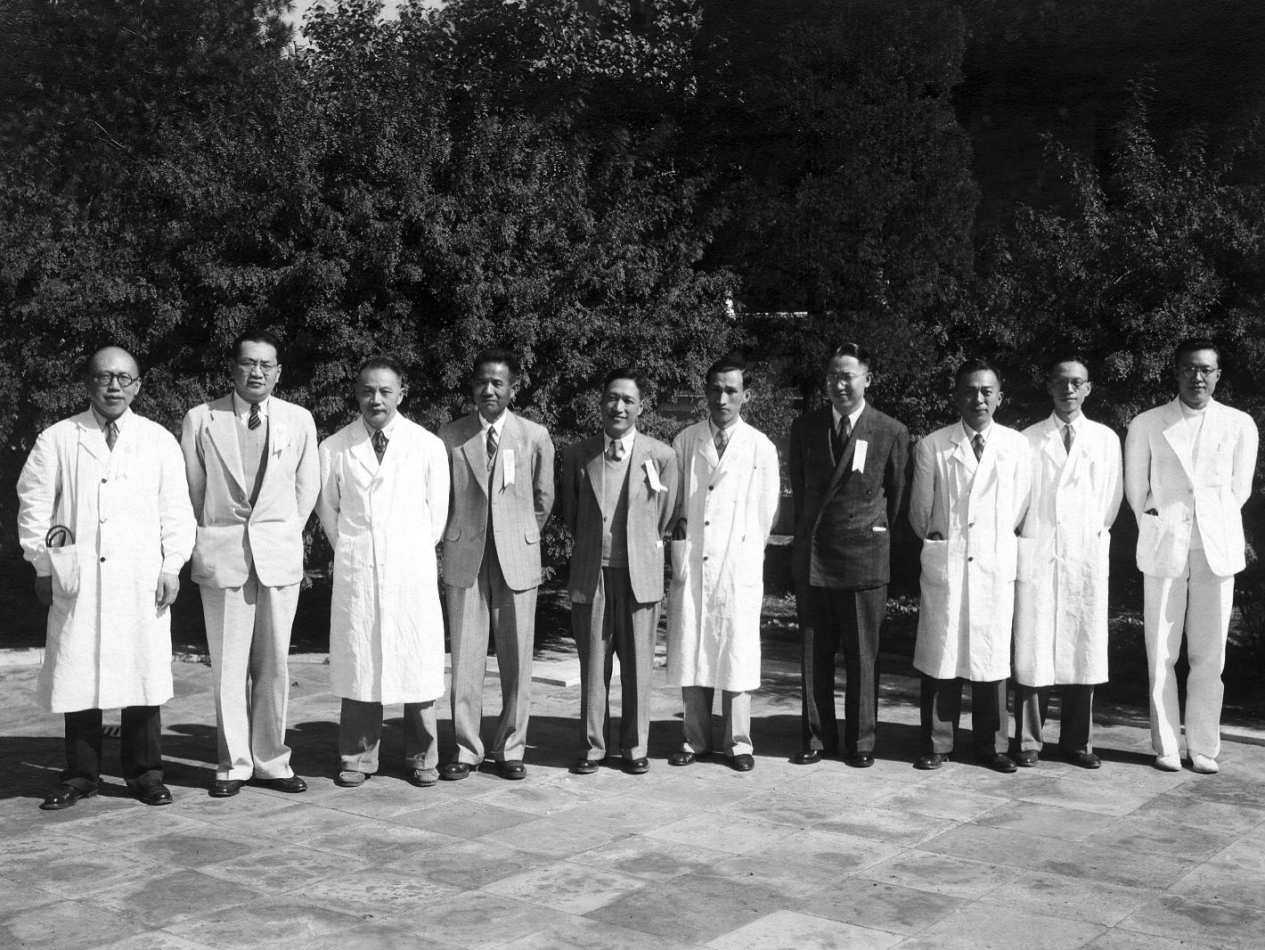
**Previous internal medicine general residents (Oct. 9th, 1949)**. From left to right: Xiaoqian Zhang, Shihao Liu, Shaowen Xie, Chaohong Wu, Xianyi Zhu, Jiadong Deng, Wanson Ma, Guiqing Zhu, An Zhang, Qi Fang

After the founding of New China, Dr. Zhang, the dean of Hsiang-Ya Medical School for 11 years, was invited to return to Peking Union Medical College Hospital again to assume the rehabilitation of internal medicine. Under his inspiration, many elites from the south area and even overseas gathered at PUMCH with the same mission (Fig. 2). He took charge of the establishment of the professional groups of digestion, infectious diseases, blood, respiration, immunity, genetic disease and so on; He wrote to the Central Government to resume the eight-year medical education of Peking Union Medical College. He restored the PUMCH’s old traditions like the resident system and ward rounds; he developed the disciplined planning of the internal medicine and improved the medical education system for PUMCH.

**Figure 3:**
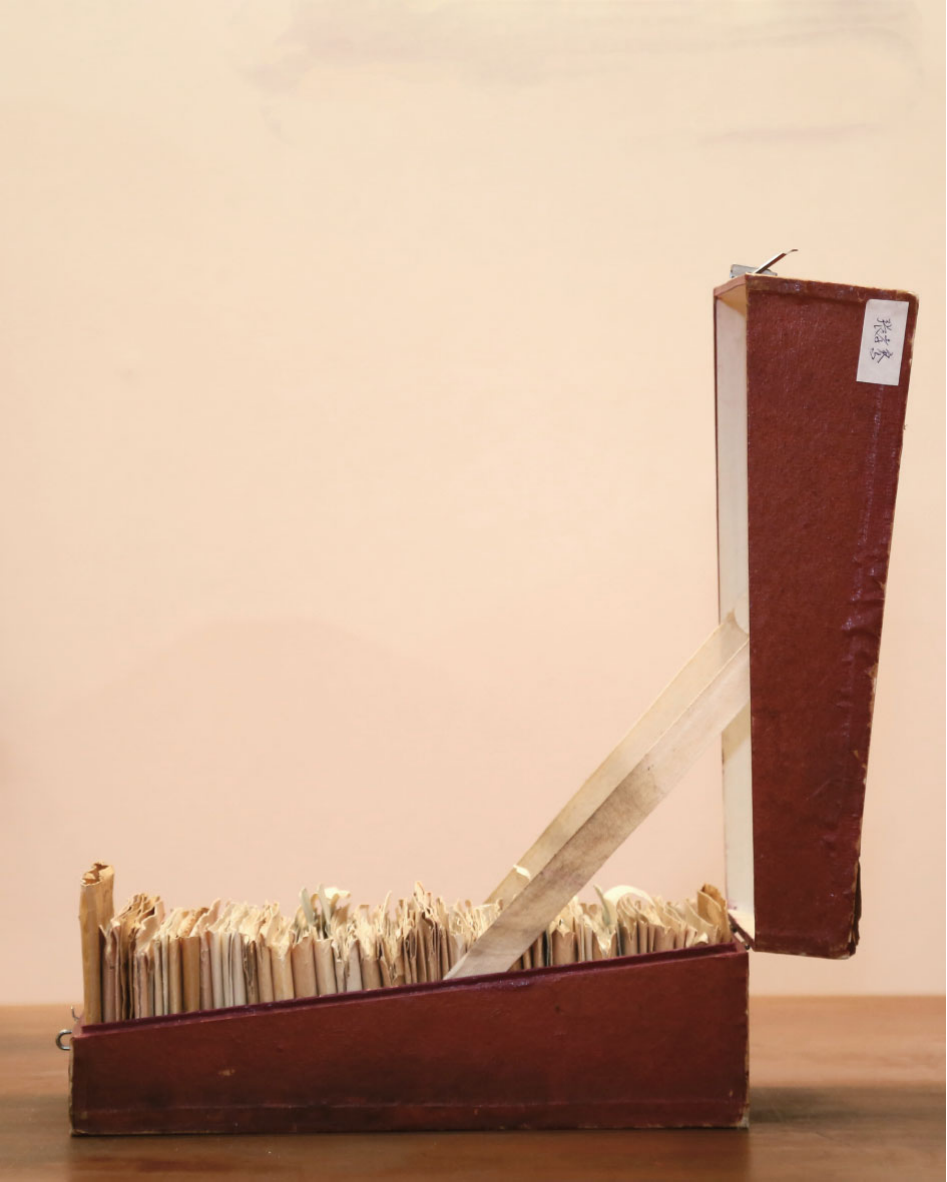
**Dr. Xiaoqian Zhang’s Notes**

PUMCH has always attached great importance on clinical practice which was also what Dr. Zhang insisted on. He devoted all his life to the clinical practice of internal medicine and made many achievements in the field of internal medicine. When it comes to the diagnoses for patients, Dr. Zhang could always hit the mark when opinions varied and could recall every symptom of a patient accurately even after quite a long period which relied on his special “notes” to a great extent. He always carried a small notebook during outpatient service or ward rounds and would record everything he deemed important or doubtful (Fig. 3). He believed that clinical issue could be well completed only when one could analyze specific issue case by case without subjectivity or arbitrariness. Every word in his small notebooks is the culmination of his painstaking effort; each sentence may retrieve life from the verge of death (Zhang, 1984).

In the middle of 1960s, a female patient who would shock once catching a cold came to PUMCH. Dr. Zhang had a familiar feeling with the patient and asked about her medical history and found out that she had been hospitalized in PUMCH due to dystocia hemorrhage 30 years ago. However, the original medical records and data were completely gone because of war. After thoroughly digging into a pile of his old special “notes” and fragments of memories,he was suddenly enlightened. The original hemorrhage caused necrosis of the pituitary gland, leading to hypopituitarism, resulting in deficiencies of endocrine thyroid glands, adrenal glands and lacks in emergency response. When the patient was subjected to an emergency infection, the shock would occur. Based on this, she was diagnosed as Sheehan’s syndrome. She was given thyroid and adrenal cortex hormones as an alternative treatment, and then her condition improved quickly (PUMCH, 1988).

Many of his lifelong mottoes has been praised until now like “Tread, as if on the thin ice; Walk, as if on the brink of a deep gulf” (Ge, 2016). In the winter of 1981, he received a medical record requiring him to give a written consultation, and the patient was a farmer. After reading the medical records, he demanded the bone marrow aspiration and lymph node examination for the patient. Two days later, having not received the results of the tests, Dr. Zhang, though in his 80s, went to visit the patient in person regardless of the distance and severe coldness. He had a particular affection on the patients, even in his late years. One night in March 1987, Dr. Zhang who just weakly woke up in the ward, asked for the vice director of the internal medicine department to come to his bed. He cast his doubt directly once he saw the vice director: “The hospital is so large that there are so many patients. Can they find a doctor at night in an emergency?” After getting a satisfactory answer, he fell asleep without any burden. Stories like these are too numerous to enumerate. Medical ethics and medical skill were always completely unified for him. “I would die before I give up” was Dr. Zhang’s life philosophy all along (PUMCH and Xiangya Hospital of Central South University, 2008).

Dr. Zhang was erudite in medical knowledge and clinical experience and was well known for his profound theoretical foundation, but he never slacked in the pursuit of advanced knowledge and studied sedulously during his lifetime. He spent almost every Sunday morning in the library. After cataract surgery, he wanted to read once seeing things. In case other people might stop him, he ordered a car to the library by himself regardless of his ophthalmologist’s dissuade. When he was caught by others, he laughed and said humorously, “it seems that one cannot do anything wrong but you know I haven’t read books for such a long period”. On that day, he kept reading until the closed bell rang for several times. When Dr. Zhang was already an octogenarian, his students advised him not to go to the library on Sunday. He replied, “medical science nowadays is developing so fast and I could hardly catch up with it without continued learning” (PUMCH, 1988).

On August 8th, 1987, Dr. Zhang passed away in Beijing, at the age of 90. There were two regrettable things for him: one was “the Clinical Supplements” had just started; the other was his desire of continuing outpatient service until the age of 90 never came true. As what was written in “Hippocrates: The Oath of Medicine”: “In every house where I come I will enter only for the good of my patients, keeping myself far from all intentional ill-doing and all seduction and especially from the pleasures of love with women or with men, be they free or slaves”. Dr. Zhang used “caution, prudence, dread and fear” as his life motto and carried out the oath all along (Zhang, 1982).

**Figure 4:**
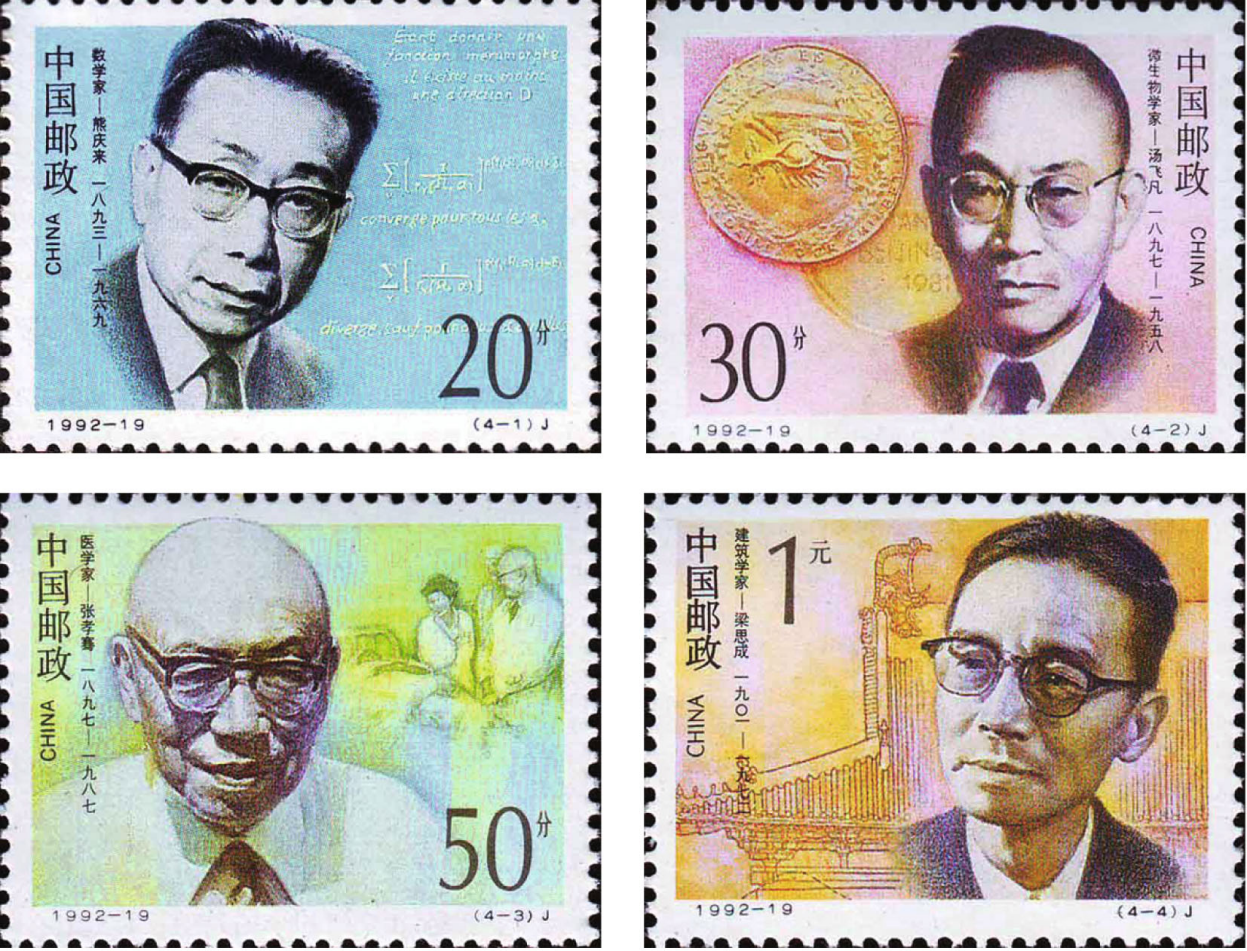
**“Modern Chinese Scientists (3rd series)” stamps**. The Ministry of Posts and Telecommunications of P.R.C. issued on Nov. 20th, 1992, a set of four commemorative stamps entitled “Modern Chinese Scientists (3rd series)” with a total face value of RMB 2.00. The stamps are respectively: Qinglai Xiong, mathematician; Feifan Tang, microbiologist; Xiaoqian Zhang, physician and Shih Cheng Liang, architect

He devoted all himself to save the nation via medical science; he paid close attention to people’s livelihood and regarded the patients as his “teacher” (Ge, 2016); he was the modern medical pioneer, but never failed to remember that “I’m a doctor”. He is not only a mere doctor but also a monument that has stretched over three centuries that deeply influence the contemporary society (Fig. 4).

A hundred and twenty years later, Dr. Zhang’s edification, forthright admonition and his voice and expression still emerge in our mind. China has been undergoing a radical change after Dr. Zhang’s period, and PUMCH, the hospital he dedicated his whole life to, is now also heading into a new era. China has already grown up with a more advancing healthcare system and healthier people which were just the destination Dr. Zhang had been struggled for throughout his life (Zhang, 1983).
